# HyLiTE: accurate and flexible analysis of gene expression in hybrid and allopolyploid species

**DOI:** 10.1186/s12859-014-0433-8

**Published:** 2015-01-16

**Authors:** Wandrille Duchemin, Pierre-Yves Dupont, Matthew A Campbell, Austen RD Ganley, Murray P Cox

**Affiliations:** Statistics and Bioinformatics Group, Institute of Fundamental Sciences, Massey University, Palmerston North, New Zealand; Present address: Laboratoire de Biométrie et Biologie Évolutive, UMR CNRS 5558, Université Lyon I, Villeurbanne, F-69622 France; Institute of Mathematical and Natural Sciences, Massey University, Auckland, New Zealand

**Keywords:** Hybrid, Allopolyploid, Homeolog, RNA-seq, Read assignment

## Abstract

**Background:**

Forming a new species through the merger of two or more divergent parent species is increasingly seen as a key phenomenon in the evolution of many biological systems. However, little is known about how expression of parental gene copies (homeologs) responds following genome merger. High throughput RNA sequencing now makes this analysis technically feasible, but tools to determine homeolog expression are still in their infancy.

**Results:**

Here we present HyLiTE – a single-step analysis to obtain tables of homeolog expression in a hybrid or allopolyploid and its parent species directly from raw mRNA sequence files. By implementing on-the-fly detection of diagnostic parental polymorphisms, HyLiTE can perform SNP calling and read classification simultaneously, thus allowing HyLiTE to be run as parallelized code. HyLiTE accommodates any number of parent species, multiple data sources (including genomic DNA reads to improve SNP detection), and implements a statistical framework optimized for genes with low to moderate expression.

**Conclusions:**

HyLiTE is a flexible and easy-to-use program designed for bench biologists to explore patterns of gene expression following genome merger. HyLiTE offers practical advantages over manual methods and existing programs, has been designed to accommodate a wide range of genome merger systems, can identify SNPs that arose following genome merger, and offers accurate performance on non-model organisms.

**Electronic supplementary material:**

The online version of this article (doi:10.1186/s12859-014-0433-8) contains supplementary material, which is available to authorized users.

## Background

While evolution is usually a gradual process, the creation of a new species through the merger of different parent species occurs near instantaneously [1]. Although increasingly recognized as an important process in the evolution of many biological systems [2-5], how different gene copies (homeologs) are expressed following genome merger remains a major outstanding question [6,7]. Most studies have been restricted to observing just a few genes, thus limiting the ability to study interactions between competing gene regulation systems [8]. High throughput mRNA sequencing now permits whole-genome screening of hybrid and allopolyploid gene expression [9,10]. However, identifying the parental origin of mRNA reads remains challenging, especially for researchers without advanced bioinformatics skills [11].

To fill this gap, we have developed HyLiTE – *Hy*brid *Li*neage *T*ranscriptome *E*xplorer – to produce tables of homeolog expression data from raw mRNA read files in a single step. HyLiTE automatically i) maps reads to a reference genome, ii) masks gene regions with low read coverage, iii) identifies polymorphisms that are diagnostic of parental lineages, iv) classifies reads to parental types, and v) produces detailed summary reports of gene expression in both the hybrid or allopolyploid and its parent species. The final product – tables of homeolog read counts – can be used immediately for downstream analyses (such as determining differential expression between biological conditions, and between the new species and its parents).

## Implementation

The primary design directives behind HyLiTE were i) ease of use, ii) runtime efficiency, and iii) use with non-model organisms (which encompasses most hybrid and allopolyploid species). Other key features include:

- Accommodating any number of parent species (for instance, three-parent allopolyploids such as modern hexaploid wheat) [12].
- The ability to study systems with both haploid or diploid parents, thus allowing hybrids or allopolyploids with different homeolog and allelic copies.
- Using gene references from any species closely related to the study system (hybrid and allopolyploid species often lack good genome resources).
- Accommodating any number of biological replicates (and boosting SNP identification by combining information across replicates).
- Identifying new polymorphisms that have arisen within the hybrid or allopolyploid (especially important in species derived from older merger events).
- Improving SNP calling by using (optional) genomic DNA information in addition to high throughput mRNA sequences.
- Providing statistical validation of SNP calls and automatically masking ‘polymorphisms’ with low statistical support.
- An experimental feature that identifies putative chimeric genes (i.e., genes in which the homeologs have recombined within the hybrid or allopolyploid) [13], but see Additional file 1 for details on current limits of accuracy.

The standard HyLiTE analysis, which will be adequate for most users, comprises a single, short command line. However, advanced users have complete flexibility to override individual steps. For instance, by default, Bowtie2 is used for read mapping, but HyLiTE can be run with any mapping software that returns the standard SAM mapping format.

Because HyLiTE analyzes each gene independently, the software has low RAM requirements and runtime is linear with the number of genes under study. This independence between genes also allows HyLiTE to be parallelized via optional executables (see project website for details; http://hylite.sourceforge.net). HyLiTE regularly autosaves the run state, and analyses can therefore be stopped and re-started from the last checkpoint. Extensive documentation about the algorithms implemented in HyLiTE, software validation and benchmarking against alternative pipelines is provided in Additional file 1.

## Results and discussion

### Output

The main output of HyLiTE comprises a list of read counts for each homeolog in each biological replicate. Using presence and absence of diagnostic parental SNPs, reads are classified as i) derived from a given parent, ii) consistent with two or more parents (i.e., lacking diagnostic SNPs), or iii) unknown (i.e., masked due to low read coverage). The last two classes are equally uninformative for determining homeolog expression, but can distinguish whether improvements may be possible with additional sequence data (the ‘unknown’ category) or whether part of the gene is simply uninformative for ancestry (no diagnostic parental SNPs identified). Finally, each read is marked with an additional flag if one or more new SNPs are detected within the hybrid or allopolyploid.

### Software comparison

A major point of difference between HyLiTE and alternative approaches (e.g., PolyCat [14]) is its robust statistical assessment of SNP calls and automatic masking of ‘polymorphisms’ with low statistical support. Due to the substantial error rate of high throughput sequencing technologies, sequencing errors can easily be confused with genuine polymorphisms in genes with low expression (and hence, low read coverage). The probability that a polymorphism at any given nucleotide position is a SNP rather than an error is given by a binomial distribution conditioned on the coverage level. Nucleotides with coverage less than this threshold are masked, but because coverage varies widely across even a single gene, typically only small, uninformative regions of any given gene are masked. This ‘dynamic masking’ substantially improves the accuracy with which reads are assigned to homeologs for genes with low to moderate expression. Detection of expression levels can be improved further by including genomic DNA reads due to the accuracy this imparts to SNP calling (see Additional file 1 for details).

### Worked examples

**Figure 1:**
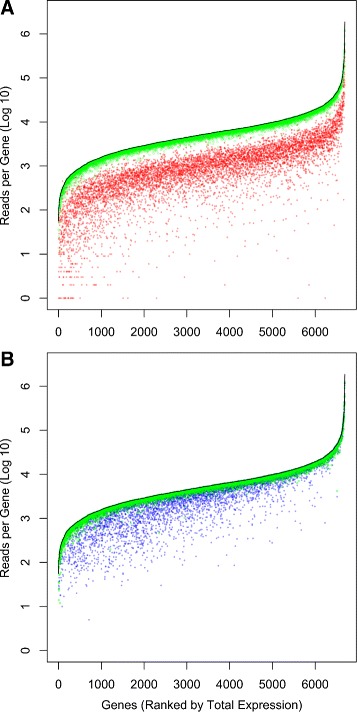
**Comparison between HyLiTE and A) the results of the Cox** ***et al.*** **study [** 9 **] and B) PolyCat [** 14 **] for** ***Epichloë*** **fungal data.** The black lines indicate the total number of reads that map to each gene, ranked by expression level. Green points indicate the number of reads assigned to homeologs by HyLiTE. Red points in **A)** indicate the number of reads assigned to homeologs in the Cox *et al.* study, while blue points in **B)** indicate the number of reads assigned to homeologs by PolyCat. The substantial improvement in read assignment by HyLiTE was obtained using its default settings.

*Fungi*. Species in the fungal genera *Epichloë* and *Neotyphodium*, already well known for their symbiotic relationships with grasses in temperate pastoral systems, are increasingly becoming the dominant model system for studying genome merger in fungi [9,15,16]. The most well studied example is Lp1, an economically important allodiploid asexual species formed from the union of a haploid sexual species, *E. typhina*, and a haploid asexual species, *N. lolii* (∼5% divergence). As HyLiTE had not yet been developed, the Cox *et al.* study instead applied a two-reference approach: gene references were generated separately for *E. typhina* and *N. lolii* using ancestry informative SNPs, and homeolog expression was then ascertained via high stringency mapping. Although estimates of gene expression are highly correlated (*r*=0.83,*P*≪0.0001), HyLiTE assigns an average of five times as many reads to homeologs as the two-reference approach, an improvement almost entirely due to reduced gene masking (Figure 1A). 86% of reads are assigned to homeologs, with the remainder classified as parental uninformative or unknown. PolyCat [14] assigned fewer reads to homeologs (Figure 1B), particularly for genes with low to moderate expression (see Additional file 1 for details).

*Plants*. To show application to a plant system, we also analyzed gene expression in a natural cotton allotetraploid, *Gossypium hirsutum*, together with diploid representatives of the A (*G. arboreum*) and D (*G. raimondii*) genomes (∼3% divergence) [10]. Assignment accuracy was tested by classifying known reads from the two diploid species. HyLiTE assigned reads to homeologs with a very low error rate (1.6%; see Additional file 1 for details). It also identified 46,206 new SNPs specific to *G. hirsutum*.

*Animals*. Finally, we analyzed gene expression in a synthetic allotetraploid fish derived from diploid goldfish (*Carassius auratus*) and diploid common carp (*Cyprinus carpio*) (∼6% divergence) (NCBI BioProject accession number: PRJNA82763). The very small number of reads available per gene (an average of only 15) caused HyLiTE to reject most SNP calls and therefore classify the majority of reads as parentally uninformative. However, the reads for which sufficient information was available to assign parental ancestry showed a very low error rate (0.22%).

## Conclusions

The formation of a new species from the merger of two or more different parent species is important in the evolutionary history of many eukaryotic lineages. Hybrid and allopolyploid species carry multiple copies of each gene (homeologs), and while homeolog expression levels can be determined from high throughput RNA sequence data, assigning reads is extremely challenging. Here, we have developed HyLiTE to automate the process of moving from raw mRNA sequence files to tables of homeolog expression in a hybrid or allopolyploid and its parent species. This single-step analysis is specifically designed for ease-of-use, particularly for non-computational scientists. HyLiTE therefore allows gene expression patterns to be explored on a whole-genome scale even for species with very complex patterns of genome merger.

## Availability and requirements

**Project name**: HyLiTE**Project home page**: http://hylite.sourceforge.net**Operating systems**: Linux, OS X, Windows**Programming language**: Python**Other requirements**: None**License**: GNU GPL v. 3.0**Any restrictions to use by non academics**: None

## Additional file

Additional file 1
**Algorithms, validation and benchmarking.** Documentation of algorithms, software validation and benchmarking against alternative pipelines.
