# Supplementary material for: HyLiTE: accurate and flexible analysis of gene expression in hybrid and allopolyploid species
Source: BMC Bioinformatics. 2015 Jan 16;16(1):8. doi: 10.1186/s12859-014-0433-8 (PMC4300824; doi:10.1186/s12859-014-0433-8)
Supplement: Additional file 1 — Algorithms, validation and benchmarking. Documentation of algorithms, software validation and benchmarking against alternative pipelines. [file 12859_2014_433_MOESM1_ESM.pdf]

ADDITIONAL FILE 1

HyLiTE: Supplementary Materials

Wandrille Duchemin, Pierre-Yves Dupont, Matthew A Campbell, Austen RD Ganley and Murray P Cox

1 Algorithm for Detecting SNPs

Error model

A single nucleotide mismatch in a read relative to the reference can have two causes: either a genuine polymorphism, or an error introduced by the sequencing technology.

Consider a unique ε, the probability that an error is generated on a read by the sequencing technology at a given position. Every base in a read has probability $\frac{\varepsilon}{3}$ of being an error. (The denominator is three because, for any given position, one base is the reference, while the other three bases are tested independently as being either SNPs or errors).

The distribution of errors along reads is not uniform. However, for a given coordinate in a gene (with the exception of boundary conditions at gene extremities), the corresponding position in aligned reads does tend to be uniformly distributed. Further, it is common practice to trim bases from reads when quality scores drop below some low threshold, thus guaranteeing a minimum level of sequence quality for the entire read [1]. We favor the SolexaQA package to perform this trimming (`http://solexaqa.sourceforge.net`).

It follows that the occurrence of a specific incorrect base (*i.e.,* a base that does not represent the true genotype) at a given coordinate in the reference gene follows a binomial law with parameters

$p = \frac{\varepsilon}{3}$
n = number of reads (from the same organism, but across all genomic DNA and RNA samples) mapping at that position (*i.e.,* the local coverage)

If the number of reads carrying an observed mismatch is a statistical outlier under such a binomial law, the variant can be considered a SNP rather than an error. (Note that the reverse does not necessarily hold: a gene with low expression might produce so few reads that a true SNP cannot be distinguished from sequencing error. See section 4 on the benefits of using genomic DNA reads to improve expression estimates below).

Parameter Values

Using the SolexaQA package [1], the error rate ε of the Cox *et al.* dataset [2] was determined to be 0.02. (This value is typical of current Illumina sequence datasets; unpublished results). Therefore

$$p = \frac{\varepsilon}{3} = \frac{0.02}{3} \tag{1}$$

The value n was determined as the number of reads at each coordinate along the gene (*i.e.,* the local coverage).

The statistical threshold α was determined in the following way. As testing for the presence of SNPs is performed at every coordinate in every gene, a very large number of independent statistical tests are necessarily run. Therefore, the standard statistical α (*e.g.,* 0.05 or 0.01) must be corrected for multiple testing. Because *a priori* we do not know the number of tests m that need to be performed, other than $m \gg 1$, we employ the Rough False Discovery Rate (RFDR) to correct our α in the limit $m \to \infty$

$$\alpha_{adjusted} = \frac{\alpha(m+1)}{2m} \tag{2}$$

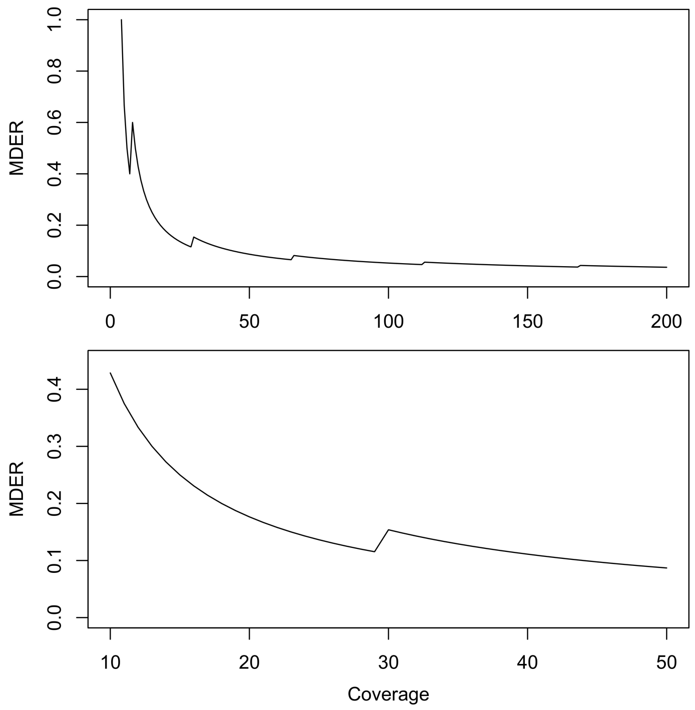

Figure S1 Decline in the Minimum Detected Expression Ratio (MDER) with increasing coverage. Upper panel: coverage from zero to 200; lower panel: enlargement of region showing coverage from 10 to 50.

and for large m

$$\alpha_{adjusted} \approx \frac{\alpha}{2} \tag{3}$$

It follows that a global probability threshold of 0.01 would require an adjusted $\alpha = 0.005$. However, we note that there are currently major concerns around statistical testing in scientific research, particularly around low P value thresholds. Therefore, following the guidelines of Johnson *et al.* [3], we advocate for a more conservative α of 0.001.

Minimum Coverage Rates

The local coverage rate n is a key parameter that allows us to distinguish between genuine SNPs and errors. As the binomial law is discrete, small values of n return probability estimates of whether a mismatch is a genuine SNP (or an error) in choppy, discrete steps (see Figure S1). Because the local coverage n changes along the gene, we have developed an algorithm that determines the lower limit of n for which the binomial law returns probabilities with poor reliability.

Consider, for example, local coverage of just two reads. The expected number of errors is effectively zero, which implies that a true error at that gene coordinate would be called as a SNP. This is obviously undesirable.

For haploid organisms, only one genotype is expected at any gene coordinate. Given binomial probabilities, coverage of three reads is sufficient to detect genuine SNPs with high reliability. If coverage is less than three reads, the gene coordinate is masked and is not used to classify reads to parental types.

For polyploid organisms, multiple genotypes are possible. Further, RNA-seq data does not guarantee equal representation of each allele (either by chance, or because the different alleles do not have the same expression). Therefore, we consider the ratio of expression between the least expressed allele and the sum of the remaining alleles. Consider, for example, that one allele in a parent is expressed ten times, while the other is expressed only once. The expression ratio would be $\frac{1}{10} = 0.1$. This lets us define the Minimum Detected Expression Ratio (MDER) as the limit below which a SNP on the least expressed allele could be detected as an error. So, for given α, p and n, the MDER corresponds to the expected occurrence of a specific error (ε_{exp}) divided by $n - \varepsilon_{exp}$

$$MDER = \frac{\varepsilon_{exp}}{n - \varepsilon_{exp}} \tag{4}$$

Figure S1 (upper panel) shows the improvement in MDER with increasing coverage. (The limit tends to p as $n \to \infty$). Note that the 'choppy' curve results from the discrete nature of the binomial law. In short, however, the higher the coverage, the better a large difference in expression level between parental alleles can be distinguished. Figure S1 (lower panel) shows an enlargement of the upper panel, with a focus on coverage levels that are currently in a more cost effective range.

At 20-fold coverage, the MDER is 0.18 (exactly $\frac{3}{17}$), which only improves (*i.e.,* declines) as the coverage increases. We propose this value as a minimum coverage level for polyploid species. Put differently, an MDER greater than or equal to $\frac{3}{17}$ (*i.e.,* a minor allele frequency greater than or equal to 0.15) will always allow SNPs to be detected with high reliability if the coverage level is greater than or equal to 20. Under default settings, gene coordinates with coverage lower than 20 reads are masked. Both of these thresholds (haploid and polyploid) can be changed by the user.

Implementation

With the statistical framework for SNP detection in place, describing the SNP calling algorithm implemented in HyLiTE is relatively straightforward. At a given gene coordinate for a given organism:

1 Temporarily aggregate all genomic DNA and RNA reads. Thus, the local coverage becomes the sum of local coverages across the different samples.
2 Count every genotype and compare the values obtained to their expected counts under the binomial distribution.
3 Select the k best genotypes (where k is the ploidy of the organism).
4 Consider each statistically validated genotype that differs from the reference as a true SNP.

Note that temporarily combining data across samples increases the total coverage available for an organism, decreases the MDER, and therefore reduces the number of gene coordinates that need to be masked. (See section 4 on the use of genomic DNA reads to improve RNA read classification).

For example, at a given gene coordinate with reference **A**, a hybrid or allodiploid might have two biological replicates with counts:

```
sample1: A:14 T:1 G:54 C:2
sample2: A:5  T:0 G:14 C:0
```

After temporarily aggregating the two replicates, the counts become:

```
          A:19 T:1 G:68 C:2
```

The total coverage is 90 reads, which greatly exceeds the minimum suggested coverage threshold for polyploids ($n = 20$). According to the binomial distribution at this coverage level, calling a SNP requires the same mismatch to be observed in at least five reads. Therefore, only two possible genotypes remain: **A** and **G**. (Correspondingly, the observed mismatches at **T** and **C** are called as sequencing errors). Because the organism is diploid in this example, up to two possible genotypes can be present at the same gene coordinate and both of the called genotypes are retained. As **A** is the reference state, we conclude that a SNP exists with the alternative state **G**, and that both **A** and **G** states are present.

Alternative Coverage Thresholds

We show above that the default coverage thresholds used by HyLiTE offer a good trade-off between sensitivity and reasonable coverage goals. However, users are encouraged to tune these thresholds depending on their biological questions and the quality of their sequence data. This can be easily managed by changing the two options '–min_coverage_haploid' and '–min_coverage_polyploid' in the HyLiTE command line.

Users can also change the desired α value and expected error rate by employing an alternative parameter file with the command flag '–alternative_params'. The alternative parameter file would look similar to this:

```
#SNP detection
MIN_COVERAGE_HAPLOID = 3       # alternative minimum coverage for haploid organisms
MIN_COVERAGE_POLYPLOID = 20    # alternative minimum coverage for polyploid organisms
EXPECTED_ERROR_RATE = 0.02/3   # alternative total error rate
ALPHA = 0.001                  # alternative alpha value
```

2 Algorithm for Classifying Reads

The primary purpose of HyLiTE is to determine the parental origin of high throughput RNA reads from a hybrid or allopolyploid. The following sections describe how this goal is achieved.

Fingerprints

We define the sequence of SNPs present, absent or masked (due to poor coverage) at specific coordinates along a gene as a 'fingerprint'. Specifically, we distinguish two types of fingerprint:

- Parent fingerprints: where information about the presence, absence and masking of SNPs are stored, gene-by-gene, for each parent.
- Child fingerprints: where every read in the hybrid or allopolyploid has its own fingerprint, referencing presence or absence of every SNP on that specific read.

Note that child fingerprints do not allow masking. As noted above, we encourage poor quality read segments to be trimmed from the dataset (*e.g.*, using the 'DynamicTrim' function of the SolexaQA package [1]).

Read Tagging for Diploid Parents

Diploid parents can have up to two fingerprints at each SNP position to allow for allelic heterozygosity. Accounting for this heterozygosity is achieved locally through read tagging (*viz.* linkage analysis).

Read tags have three possible values: unassigned, gene copy 0, and gene copy 1. Each parent is initiated with an unassigned tag. When a heterozygous position is detected, the algorithm first looks for existing reads with an assigned tag. (Note that HyLiTE steps through each gene from beginning to end, and therefore processes multiple reads in parallel). If no earlier read has an assigned tag (as occurs when starting a new gene), gene copy 0 or 1 is assigned arbitrarily to the allele, and each subsequent read carrying that SNP variant is given

the same tag. However, if one or more reads have already been tagged (*i.e.,* they have already been assigned to an allele), HyLiTE determines which allele coincides with which tag and then propagates those earlier tags to all reads bearing the same genotype at the new gene coordinate.

Algorithm

HyLiTE detects SNPs sequentially along the gene for each organism simultaneously. This means that when HyLiTE moves to a new gene coordinate, all leftward SNPs have already been detected in both the parents and the hybrid or allopolyploid descendant. This is an important characteristic: as soon as a read from the hybrid or allopolyploid has been processed, all SNPs (present, absent and masked) have already been detected and referenced in every organism. Thus, HyLiTE can immediately classify that read.

After removing masked SNPs (*i.e.,* SNPs with poor coverage in at least one parent), the remaining SNPs can be denoted by 0s and 1s, where 0 indicates the absence of a SNP and 1 indicates its presence. The same process can be performed for SNPs in each parent.

Consequently, classification comes down to a series of comparisons between the parent and child fingerprints. For reasons of computational speed, these are treated as a list of boolean values and analyzed with bitwise operators. The process is:

1 Eliminate any child-specific SNPs, as these cannot help (but can hinder) comparison of the parent and child fingerprints. If any SNP is eliminated, set an 'N' flag showing that there is at least one 'new' non-ancestral SNP on the read.
2 For remaining SNPs, perform a bitwise XNOR operation between the child fingerprint and the fingerprint of each parent.
3 If this XNOR operation returns a list composed only of ones for any parent (*i.e.,* the fingerprints match perfectly), consider the read as coming from this parent.
4 If no perfect match is found, recursively try to 'recombine' parent fingerprints until a perfect match is found.

If multiple parents exhibit a perfect match after step 3, the read is classified as equally consistent with coming from more than one parent. For step 4, the 'crossing' operation is simply implemented as an OR operation between parent XNOR results. Scenarios that imply recombination between fewer parents are preferred.

Worked Examples

Simple Example

Consider a read r from an allodiploid with two haploid parents, P1 and P2. Let r span coordinates 35 to 124 on *gene1*. The coordinates of SNPs in this region, with absence/presence/masking information for the allodiploid read r and parents P1 and P2 is:

```
position r P1 P2
40       1  1 -1
52       1  0  0
65       1  1  1
96       1  0  1
113      1  1  1
```

Where '1' signifies the presence of the SNP, '0' signifies its absence, and '-1' signifies a masked SNP (*i.e.,* coverage falls below the allowed threshold for that organism at that gene coordinate).

The first step eliminates the masked SNP at position 40. The fingerprints for each organism then appear as follows:

```
r:  1111
P1: 0101
P2: 0111
```

The SNP at position 52 is child-specific (*i.e.,* it is found only in the allodiploid, but neither of its parents). This SNP is removed and the new SNP 'N' flag raised. The fingerprints now appear as follows:

```
r:  111
P1: 101
P2: 111
```

An XNOR operation is performed between the fingerprint of the allodiploid read *r* and the fingerprints of each of the parents:

```
r XNOR P1: 101
r XNOR P2: 111
```

In this step, *r* XNOR P2 yields a result with all values 1, while *r* XNOR P1 yields a mixture of 0s and 1s. We therefore conclude that P2 is the likely origin of the allodiploid read *r*.

The category assigned to *r* is '(P2)+N', indicating an origin in parent P2, as well as the presence of a new child-specific SNP in the allodiploid. (Note that the use of parentheses seems redundant in this simple case, but quickly becomes crucial when dealing with multiple parents).

Complex Example

Consider a read *r* from an allotriploid with three parents, P1, P2 and P3. All preliminary steps, including removal of masked and child-specific SNPs leads to the fingerprints:

```
r:  1101
P1: 1011
P2: 1110
P3: 0001
```

The XNOR operations yield:

```
r XNOR P1: 1001
r XNOR P2: 1100
r XNOR P3: 0011
```

None of these results contains only 1s , so *r* is likely a chimeric read (*i.e.,* the result of recombination event(s) between two or more parental types). First, we test for biparental crossovers by performing a bitwise OR operation between pairs of results produced by the previous XNOR operation:

Category	Unknown	*E. typhina*-like	*N. lolii*-like	*E. typhina* or *N. lolii*	*E. typhina*/*N. lolii* chimeric reads
Manual	45	76	133	0	1
HyLiTE	22	73	130	1	29

Table S1 Comparison of manual read classification versus read classification by HyLiTE. Each column corresponds to a different read class, as defined by HyLiTE. *E. typhina* and *N. lolii* are the haploid parents of the allodiploid, Lp1. Note that due to the reduced accuracy with which chimeric reads are detected, these are reported by HyLiTE as 'parentally uninformative'.

```
P1 + P2: 1101
P1 + P3: 1011
P2 + P3: 1111
```

One result yields only 1s and therefore indicates that the allotriploid read r likely derives from recombination between the sequences of parents P2 and P3. Note that the resulting classification – (P2+P3) – actually implies the bitwise operation (r XNOR P2)OR(r XNOR P3). Note that due to poor accuracy rates (see validation sections), chimeric reads are reported as 'parentally uninformative'.

3 Validating Read Classification

To determine the accuracy with which HyLiTE assigns reads to parental types, we adopted a combination of simulation and manual validation approaches. Assignment accuracy was tested for hybrids and allopolyploids with both haploid and diploid parents.

Haploid Parents

We analyzed a dataset from the fungal allodiploid system described in the main text [2]. EfM3.000420 is a gene 1.1 kb in length with regions of both good and poor coverage, thus allowing us to assess the functionality of HyLiTE in both positive and negative conditions. Real RNA reads for the gene EfM3.000420 were extracted, classified automatically with HyLiTE, analyzed manually by visualizing the reads in the Integrative Genomics Viewer (IGV) [4] and then assigned to parental types one-by-one.

Both analyses identified regions where poor coverage in one of the parent species demanded masking. This typically occurs at the ends of genes, but can occur internally as well. EfM3.000420 was masked over bases 1–242, 630–648 and 854–1101 (where coordinate 1101 is the end of the gene). Low RNA read coverage in the parent species required nearly half of the gene to be masked. (Note that genomic DNA reads would circumvent this masking problem, as described in section 4 below). 35 SNPs were detected in the remaining unmasked regions. Both HyLiTE and the manual annotation identified exactly the same set of SNPs.

These 35 SNPs were used to classify reads to parental types, as quantified in Table S1. The manual classification very closely matches the classification made by HyLiTE. The main difference is that HyLiTE classified a number of reads as chimeric (*i.e.,* recombinant reads between the *E. typhina* and *N. lolii* parent sequences). When these reads were examined further, we identified the following mitigating conditions:

- 14 reads were located close to the masked region 630–648 and showed poor quality read alignment (*i.e.,* they were artifacts of Bowtie2 mapping errors, not HyLiTE).
- 12 reads were located near a SNP found only in the *E. typhina* parent, but not in the descendant allodiploid.

The few remaining misallocated reads were present in low complexity regions where indels and sequencing errors are common. While these types of error can be identified by eye, neither the mapping software Bowtie2

nor HyLiTE can make such subtle distinctions. Features of this nature explain all of the observed differences between the manual classification and the classification of HyLiTE.

We emphasize that manual classification of this single gene was a long process (approximately half a day's work) and mentally taxing. Although manual classification of reads outperforms the computational classification of HyLiTE, the results are remarkably similar for most read classes. Of course, manual classification is simply not possible for hundreds of millions to billions of reads.

We conclude that HyLiTE performs well under good quality read alignment conditions, as is usually the case for genes. Where alignment quality decreases, HyLiTE misclassifies some reads, and genes with large numbers of putatively chimeric reads are particularly prone to error. We also emphasize that the reference gene sequences must be chosen carefully because regions of poor quality alignment will increase as the reference sequence diverges from the transcriptomes under study.

Diploid Parents

To test the accuracy of read classification for tetraploid allopolyploids with diploid parents, the same simulated data was used as described below to study the effect of genomic DNA reads on RNA read classification. Mutations were inserted randomly. Because this is simulated data, both SNPs and read coverage tend to be relatively uniform along the gene sequence. For this reason, none of the 'mapping' problems identified in the real haploid dataset above were observed.

All simulated FASTQ read files were mapped to the gene sequence using the same parameters as HyLiTE, and the resulting mappings were displayed in IGV. All SNPs were identified correctly by HyLiTE. As the origin of all mutations was known, it was relatively straightforward to classify all reads manually. As all reads contained at least one diagnostic SNP (due to the 5% divergence rate for our system), the parent and allele from which every read was derived could be identified. For these simulated data, no discrepancies were found between the manual classification and the classification of HyLiTE. We note, however, the limitations of using simulated datasets, as they typically do not exhibit the complexity of real biological data (as illustrated above).

4 Using Genomic DNA Reads to Improve RNA Read Classification

Genomic DNA reads can be used to improve the classification of RNA reads by raising the call rate of diagnostic parental SNPs. To quantify this effect, we simulated DNA and RNA read data for two systems: i) haploid parents giving rise to an allodiploid, and ii) diploid parents giving rise to an allotetrapolyploid. For the haploid parent case, we simulated a 1700 bp gene with polymorphism rates of 4.5% (*i.e.,* 1 mutation per 22 bp) for the first parent and 1% (*i.e.,* 1 mutation per 100 bp) for the second parent, thus mimicking polymorphism rates observed in the fungal allodiploid system described in the main text. For the diploid parent case, we simulated a 1700 bp gene with the same polymorphism rates, but with 60% of mutations on one allele and 40% on the other. The allopolyploid was created by merging the parent gene copies, and adding new polymorphisms in the allopolyploid at a low rate (0.25%; *i.e.,* one mutation per 400 bp on average, thus corresponding to approximately one new polymorphism per gene copy). Genomic DNA and RNA reads were created by drawing 100 nucleotide sequences from these simulated genes with random start positions and strands. These simulated reads were written to different FASTQ files (one for each of the parents and the allopolyploid). As the purpose of this simulation was not to test the mapping efficiency of Bowtie2, quality scores were arbitrarily set to 'H', corresponding to a high Illumina 1.8+ Phred+33 value. The number of reads required for each coverage level were computed using:

$$N = \frac{LC}{l} \tag{5}$$

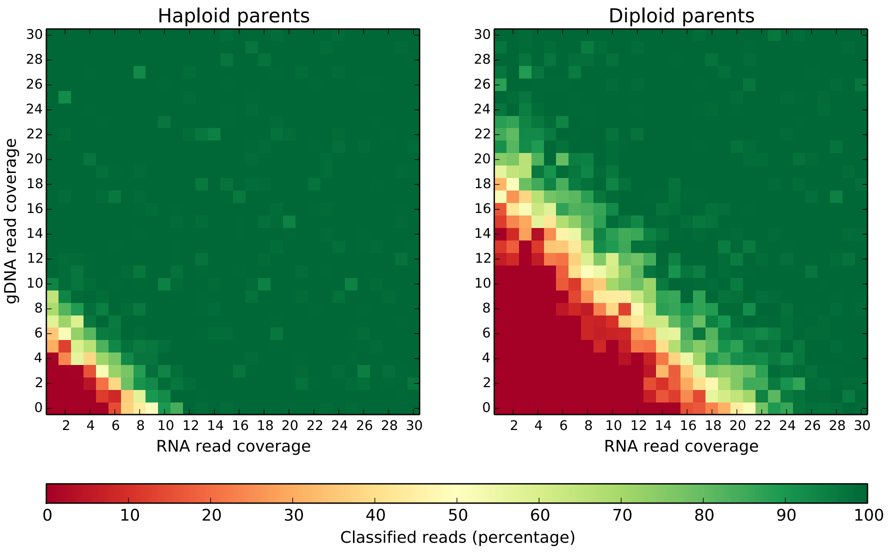

Figure S2 Improvement in RNA read classification when using genomic DNA reads. The percentage of classified reads is shown on a color scale from red (0%) to green (100%) for different genomic DNA and RNA read coverage levels.

where N is the number of reads, l is the length of the reads, L is the length of the gene and C is the desired coverage level. The percentage of reads classified by HyLiTE was determined for a range of genomic DNA and RNA coverage levels.

As expected, genomic DNA reads can improve expression estimates for genes with low to moderate expression (Figure S2). Because genes with low expression produce few reads, diagnostic parental SNPs often cannot be called, and hence, RNA reads cannot be assigned to one parent or the other. In such cases, genomic DNA reads can improve SNP calling, and thus lead to improved gene expression estimates. If no genomic DNA reads are available, expression estimates are poor for genes with less than ~10-fold RNA coverage (allodiploids with haploid parents), or ~25-fold RNA coverage (allotetraploids with diploid parents). Because RNA and DNA reads are interchangeable for SNP calling purposes, these limits also hold for genomic DNA reads: ~10-fold DNA coverage (haploid parents) or ~25-fold DNA coverage (diploid parents) is sufficient to assign nearly all RNA reads to a parental type regardless of the expression level of the gene. We emphasize that genomic DNA reads are, of course, not included in the read counts for expression estimates; they are only used for SNP calling purposes.

5 Comparing HyLiTE and PolyCat

The following comparisons employ the same datasets as in the "Worked Examples" section in the main text. In short, mRNA sequences were assigned to parental lineages (homeologs) using HyLiTE and PolyCat [5]. For comparability, the same mapping software was used with both programs, and where possible, runtime parameters were set to be as similar as possible. To illustrate its simplicity, HyLiTE was run with its default settings.

Fungi. HyLiTE results for the *Epichloë* dataset were obtained using a single command line:

```
HyLiTE -r ref.fasta -f protocol.txt -n PolyCatTest
```

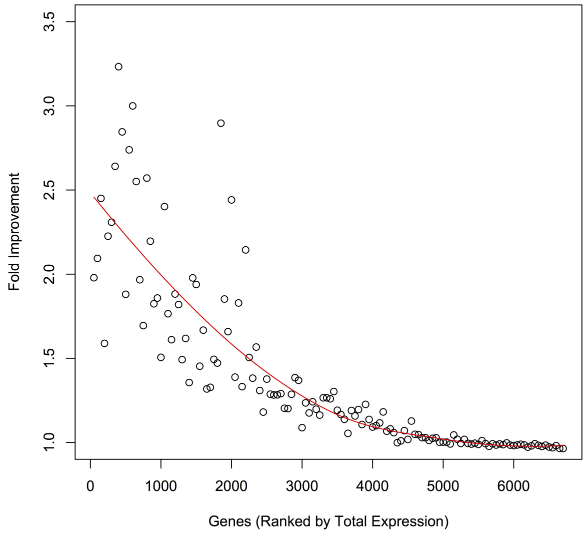

Figure S3 Median fold improvement of read assignment by HyLiTE compared to PolyCat relative to gene expression level for the *Epichloë* dataset. Fold improvement is plotted for sets of 50 genes ranked from low to high expression (points, left to right). The red line shows the local regression to the mean.

PolyCat results were obtained using the following pipeline:

1 Map reads to reference genes using Bowtie2 [6] separately for both of the parent species (*E. typhina* and *N. lolii*) and the allodiploid Lp1.
2 Create, sort and index *.bam files using SAMtools [7] separately for both the parent species and the allodiploid Lp1.
3 Run InterSNP (a companion program to PolyCat required to build the SNP index) separately on *.bam files from both the parent species.
4 Run PolyCat on the *.bam file from the allodiploid species Lp1.
5 Determine read counts from multiple output *.bam files using SAMtools and custom grep commands.

Steps 1, 2, 4 and 5 have analogs in HyLiTE, but are performed automatically. In addition, PolyCat requires a SNP index to be built for the parent species (step 3). Once built, this index can be used for multiple experiments. HyLiTE does not require a separate SNP index, and instead identifies this information gene-by-gene automatically.

The number of reads assigned to homeologs for each gene is shown in Figure 1 in the main text. HyLiTE determined homeolog expression for 6,693 of 6,694 genes in the reference (99.99%), compared to 6,638 genes in the Cox *et al.* study [2] (99.16%) and 5,995 genes for which homeolog expression was determined by PolyCat (89.56%). Although PolyCat determined homeolog expression for fewer genes than the two-reference mapping approach [2], homeolog assignment rates were substantially improved for those genes that were called (compare panels A and B in Figure 1 in the main text). Consequently, we suggest that specific software solutions (such as HyLiTE or PolyCat) should be strongly favored over alternative manual approaches.

In a direct comparison, HyLiTE assigned more reads to homeologs than PolyCat (the thin band of green points versus the cloud of blue points in Figure 1B in the main text). The number of reads assigned by HyLiTE

approached the theoretical maximum (indicated by the black line) across the full range from low to high expression. Importantly, the rate at which HyLiTE assigned reads to homeologs has very low variance across this entire expression range (*i.e.,* the green 'band' has approximately equal width regardless of the expression level). Conversely, PolyCat was much more dependent on gene expression level (Figure S3). HyLiTE assigned a median of 2.5 times as many reads to homeologs than PolyCat for genes with very low expression (and in the case of some individual genes, as much as an order of magnitude more). However, HyLiTE and PolyCat assigned almost the same number of reads to homeologs for genes with high expression (with a slight bias in favor of PolyCat for highly expressed genes).

Plants. To show application to a plant system, we also analyzed gene expression in a natural cotton allotetraploid, *Gossypium hirsutum*, together with two diploid parent genome-type representatives, *G. arboreum* and *G. raimondii* [8]. HyLiTE and PolyCat [5] assigned reads to homeologs at comparable rates, with PolyCat assigning more reads (Figure S4). Assignment accuracy was tested by classifying known reads from the parent species:

$$error\ rate = \frac{misassigned}{misassigned + correctly\ assigned} \tag{6}$$

PolyCat exhibited a higher proportion of reads (8.0%) that were incorrectly assigned than HyLiTE (1.6%). This difference seems to reflect i) poor identification of chimeric reads (which we propose above mostly results from mapping errors) and ii) alternative choices in SNP calling strategies. Of 147,453 total SNPs, PolyCat treats 23,208 SNPs (16%) as having fixed differences between the parents (say, A versus G), and therefore uses these markers to classify the parental origin of reads. In contrast, HyLiTE recognizes these positions as polymorphic in at least one of the parent species (say, A+G versus A), and therefore masks the shared state (here, A) as uninformative for read classification. Manual screening on a subset of these SNPs confirmed that most are genuinely polymorphic. Consequently, while HyLiTE assigns fewer reads, it does so with greater accuracy.

We also note that the PolyCat software was validated on a dataset containing 1,140,550,335 reads for the first parent (*G. raimondii*) and 4,070,680,434 reads for the second parent (*G. arboreum*). In contrast, the analyses described here were performed on datasets that are two orders of magnitude smaller, and therefore directly comparable to most hybrid and allopolyploid studies.

Animals. Finally, we analyzed gene expression in a synthetic allotetraploid fish derived from diploid goldfish (*Carassius auratus*) and diploid common carp (*Cyprinus carpio*) (NCBI BioProject accession number: PRJNA82763). This dataset employed the 454 sequencing technology. As with the cotton example, PolyCat assigned more reads, but also showed a higher error rate (32%) than HyLiTE (0.22%), with most errors due to incorrectly called chimeric reads. The very small number of reads available per gene (an average of only 15) caused HyLiTE to reject most SNP calls and therefore classify the majority of reads as parentally uninformative. While HyLiTE consequently assigned many fewer reads to homeologs, the proportion of misassigned reads was nearly 150 times lower.

We note that PolyCat incorrectly assigned many reads as chimeric (295,538), although this feature appeared to validate well on their original cotton example [5]. Excluding chimeric reads, misalignments still result in an error rate $\sim$5 times greater than HyLiTE. Consequently, PolyCat, which was developed and validated on the model system cotton [5], appears to perform less well on non-model systems or alternative data types.

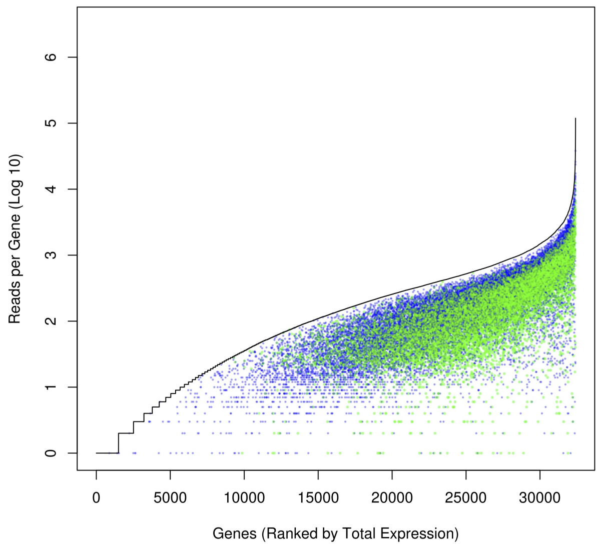

Figure S4 Comparison between HyLiTE and PolyCat for plant cotton data. The black line indicates the total number of reads that map to each gene, ranked by expression level. Green points indicate the number of reads assigned to homeologs by HyLiTE, while blue points indicate the number of reads assigned to homeologs by PolyCat. Default settings were used for both programs. Note that although PolyCat assigns more reads than HyLiTE, the number of misassigned reads is also substantially higher.

References

1. Cox, M., *et al.*: SolexaQA: At-a-glance quality assessment of Illumina second-generation sequencing data. BMC Bioinf **11**, 485 (2010)
2. Cox, M.P., *et al.*: An interspecific fungal hybrid reveals cross-kingdom rules for allopolyploid gene expression patterns. PLoS Genet **10**, 1004180 (2014)
3. Johnson, V.E.: Revised standards for statistical evidence. Proc Natl Acad Sci USA **110**, 19313–19317 (2013)
4. Thorvaldsdóttir, H., *et al.*: Integrative genomics viewer (IGV): High-performance genomics data visualization and exploration. Briefings Bioinf **14**, 178–192 (2013)
5. Page, J.T., *et al.*: PolyCat: A resource for genome categorization of sequencing reads from allopolyploid organisms. G3 **3**, 517–525 (2013)
6. Langmead, B., Salzberg, S.: Fast gapped-read alignment with Bowtie 2. Nat Methods **9**, 357–359 (2012)
7. Li, H., *et al.*: The sequence alignment/map format and SAMtools. Bioinformatics **25**, 2078–2079 (2009)
8. Yoo, M.-J., *et al.*: Homoeolog expression bias and expression level dominance in allopolyploid cotton. Heredity **110**, 171–80 (2013)
